# Supplementary material for: A genome-wide meta-analysis identifies 50 genetic loci associated with carpal tunnel syndrome
Source: Nat Commun. 2022 Mar 24;13:1598. doi: 10.1038/s41467-022-29133-7 (PMC8948232; doi:10.1038/s41467-022-29133-7)
Supplement: Supplementary file 1 — Supplementary Information [file 41467_2022_29133_MOESM1_ESM.pdf]

Supplementary information

A genome-wide meta-analysis identifies 50 genetic loci associated with carpal tunnel syndrome

Astros Th. Skuladottir, Gyda Bjornsdottir, Egil Ferkingstad, Gudmundur Einarsson, Lilja Stefansdottir, Muhammad Sulaman Nawaz, Asmundur Oddsson, Thorunn A. Olafsdottir, Saedis Saevarsdottir, G. Bragi Walters, Sigurdur H. Magnusson, Anna Bjornsdottir, Olafur A. Sveinsson, Arnor Vikingsson, Thomas Folkmann Hansen, Rikke Louise Jacobsen, Christian Erikstrup, Michael Schwinn, Søren Brunak, Karina Banasik, Sisse Rye Ostrowski, Anders Troelsen, Cecilie Henkel, Ole Birger Pedersen, DBDS Genetic Consortium, Ingileif Jonsdottir, Daniel F. Gudbjartsson, Patrick Sulem, Thorgeir E. Thorgeirsson, Hreinn Stefansson, and Kari Stefansson

Contents

Supplementary Figure 1. Regional plots of the loci associating with CTS 2

Supplementary Figure 2. Manhattan plot for individual datasets 16

Supplementary Figure 3. Correlation of effects 17

Supplementary Figure 4. Genetic propensity for CTS in different groups 18

Supplementary Figure 5. Mendelian randomization 19

Supplementary Table 1. Demographics of the carpal tunnel syndrome datasets 20

Supplementary Table 2. Weighted genome-wide significance thresholds 21

Supplementary Table 3. Mendelian randomization 22

Supplementary Figure 1. Regional plots of the loci associating with CTS

Variants are colored by the degree of correlation (r^2) with the lead variant, which is colored black. Functional variants have a squared (moderate impact) or a diamond shape (high impact). The $-\log_{10}P$-values on the left y-axis (two-sided logistic regression) are plotted for each variant against their chromosomal position (x axis). The right y-axis shows calculated recombination rates based on the Icelandic data at the chromosomal location, plotted as solid black lines. *P*-values are two-sided and derived from a likelihood-ratio test.

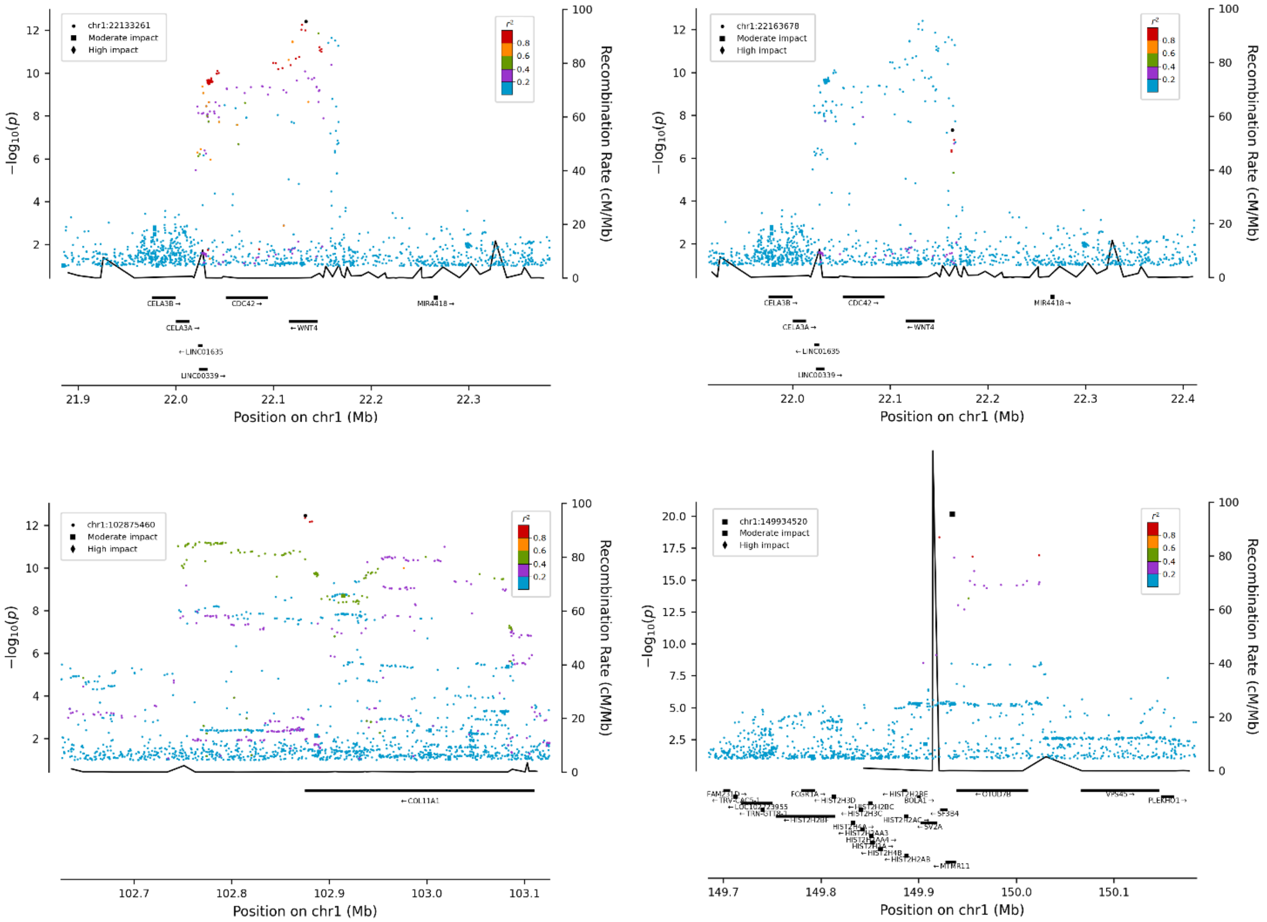

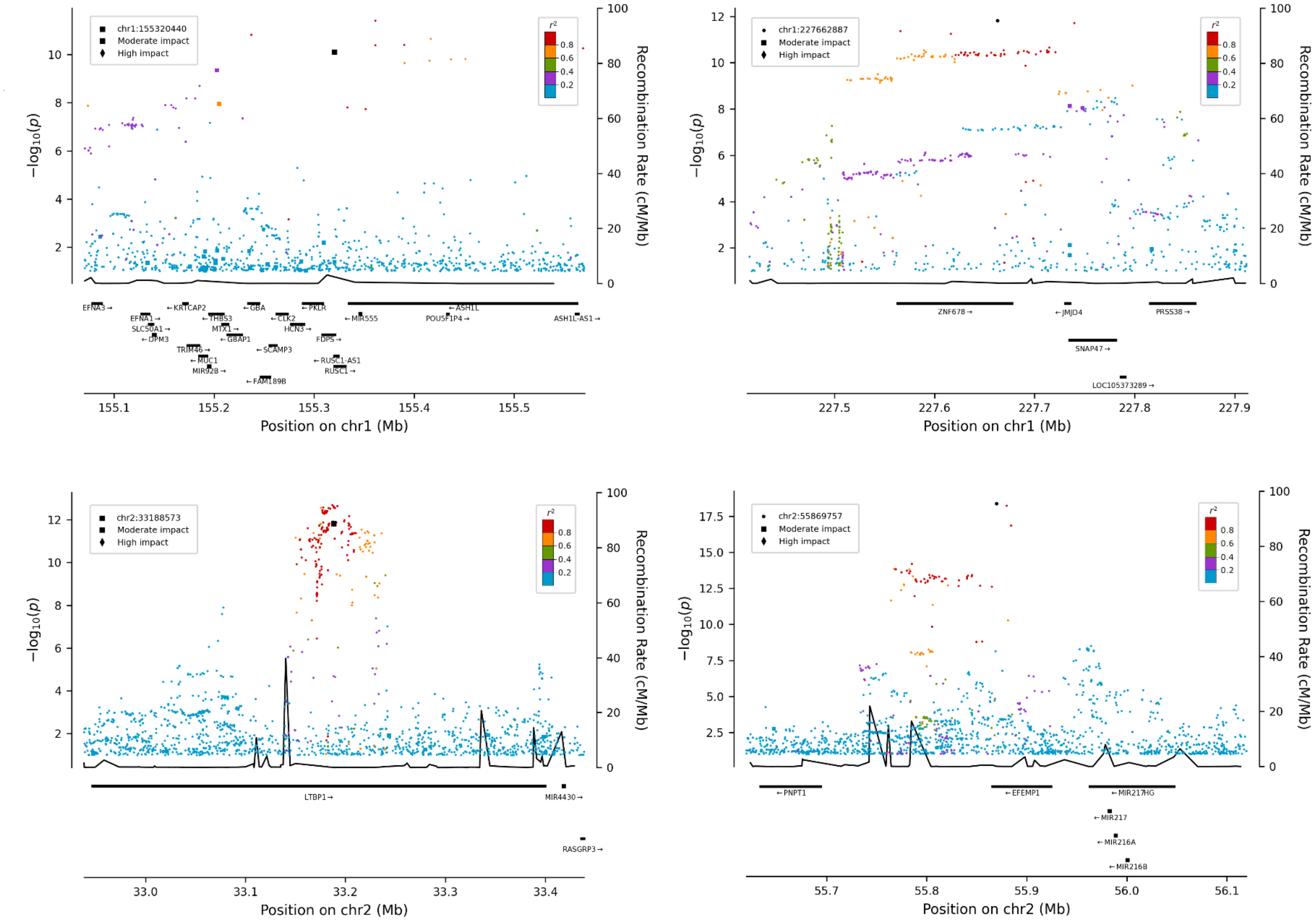

chr1:155320440
Moderate impact
High impact
r^2
$-\log_{10}(p)$
Recombination Rate (cM/Mb)
Position on chr1 (Mb)
EFNA3→
EFNA1→
SLC50A1→
←DPM3
←KRTCAP2
TRIM46→
←MUC1
MIR92B→
←THBS3
MTX1→
←GBAP1
←GBA
←SCAMP3
←FAM189B
←CLK2
HCN3→
←PKLR
FDPS→
←RUSC1-AS1
RUSC1→
←MIR555
←ASH1L
POU5F1P4→
ASH1L-AS1→
chr1:227662887
Moderate impact
High impact
$-\log_{10}(p)$
Recombination Rate (cM/Mb)
Position on chr1 (Mb)
ZNF678→
←JMJD4
SNAP47→
PRSS38→
LOC105373289→
chr2:33188573
Moderate impact
High impact
$-\log_{10}(p)$
Recombination Rate (cM/Mb)
Position on chr2 (Mb)
LTBP1→
MIR4430→
RASGRP3→
chr2:55869757
Moderate impact
High impact
$-\log_{10}(p)$
Recombination Rate (cM/Mb)
Position on chr2 (Mb)
←PNPT1
←EFEMP1
←MIR217HG
←MIR217
←MIR216A
←MIR216B

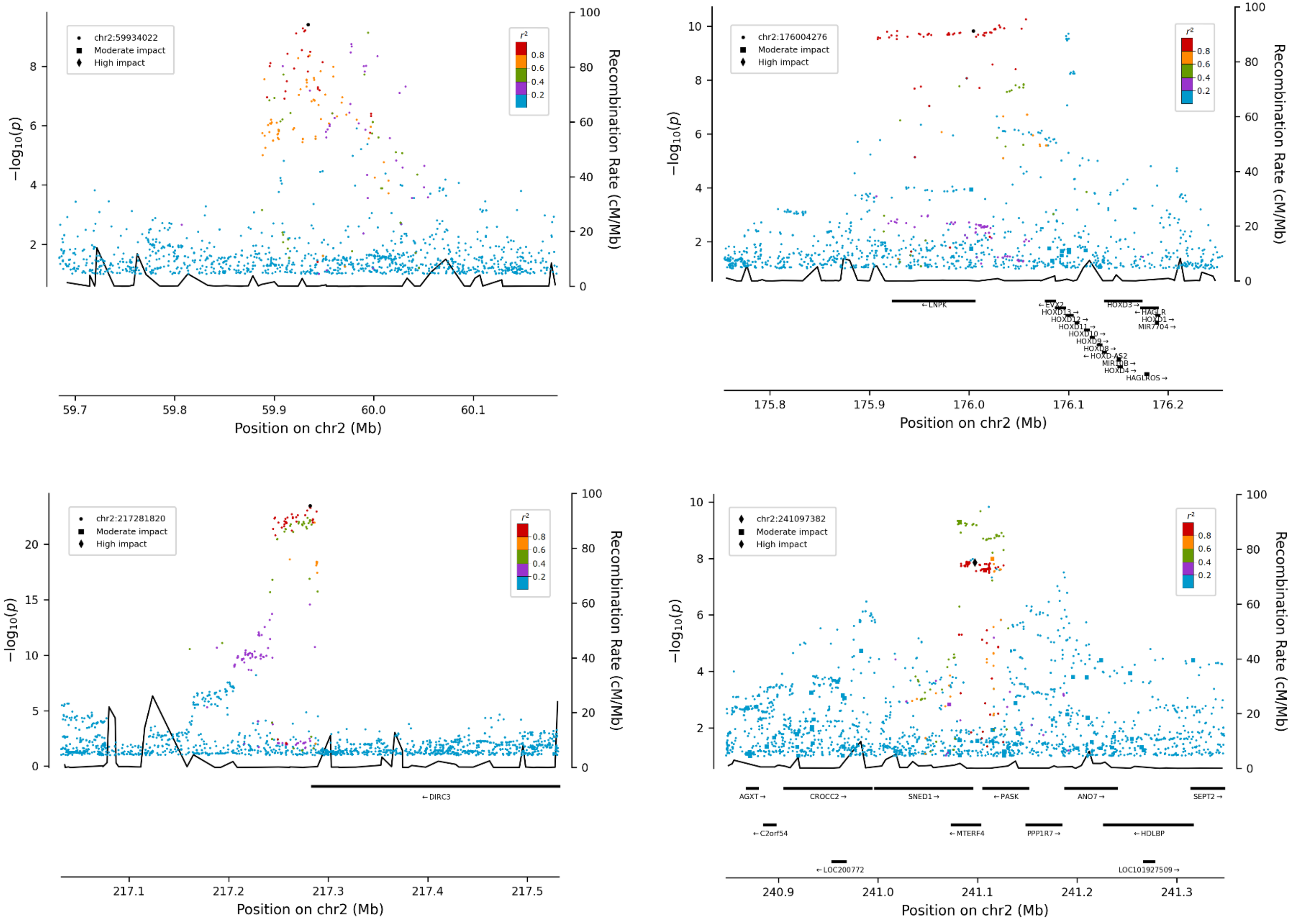
chr2:59934022
Moderate impact
High impact
r^2
0.8
0.6
0.4
0.2
$-\log_{10}(p)$
Recombination Rate (cM/Mb)
Position on chr2 (Mb)
59.7
59.8
59.9
60.0
60.1
chr2:176004276
←LNPK
HOXD3→
←HAGLR
HOXD1→
MIR7704→
HOXD13→
HOXD12→
HOXD11→
HOXD10→
HOXD9→
HOXD8→
←HOXD-AS2
HOXD4→
HAGLROS→
175.8
175.9
176.0
176.1
176.2
chr2:217281820
←DIRC3
217.1
217.2
217.3
217.4
217.5
chr2:241097382
AGXT→
CROCC2→
SNED1→
←PASK
ANO7→
SEPT2→
←C2orf54
←MTERF4
PPP1R7→
←HDLBP
←LOC200772
LOC101927509→
240.9
241.0
241.1
241.2
241.3

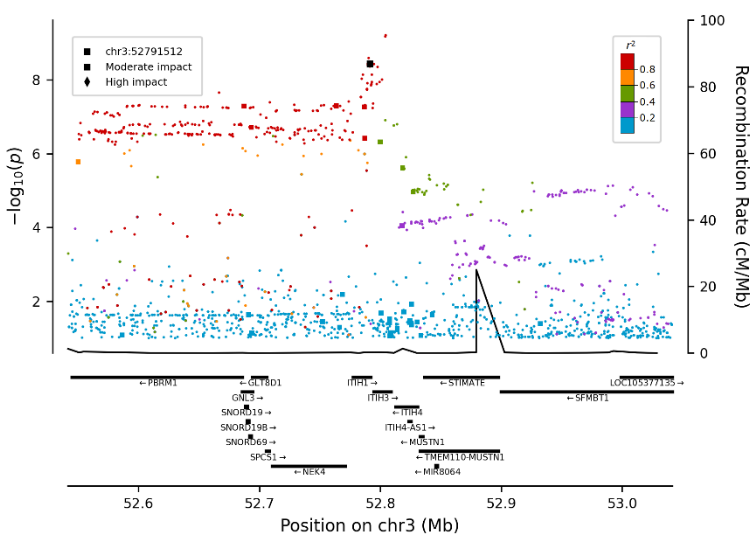
chr3:52791512
Moderate impact
High impact
r^2
0.8
0.6
0.4
0.2
$-\log_{10}(p)$
Recombination Rate (cM/Mb)
←PBRM1
←GLT8D1
GNL3→
SNORD19→
SNORD19B→
SNORD69→
SPCS1→
←NEK4
ITIH1→
ITIH3→
←ITIH4
ITIH4-AS1→
←MUSTN1
←TMEM110-MUSTN1
←MIR8064
←STIMATE
←SFMBT1
LOC105377135→
52.6
52.7
52.8
52.9
53.0
Position on chr3 (Mb)

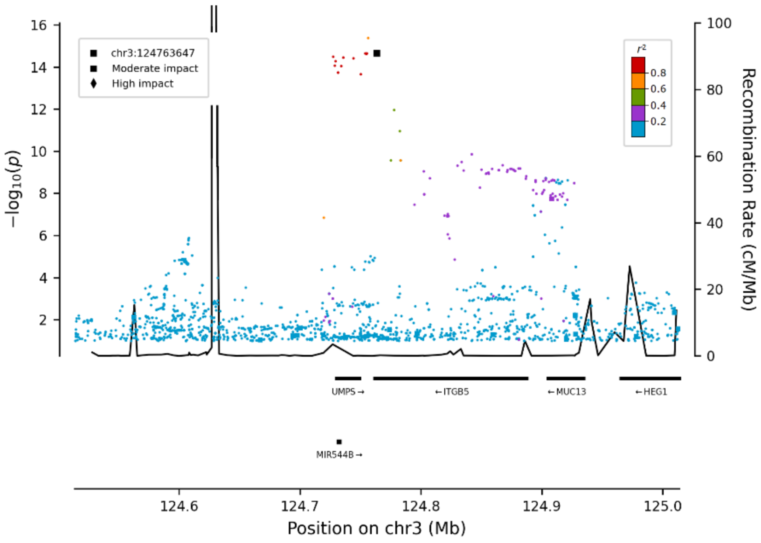
chr3:124763647
Moderate impact
High impact
r^2
0.8
0.6
0.4
0.2
$-\log_{10}(p)$
Recombination Rate (cM/Mb)
UMPS→
←ITGB5
←MUC13
←HEG1
MIR544B→
124.6
124.7
124.8
124.9
125.0
Position on chr3 (Mb)

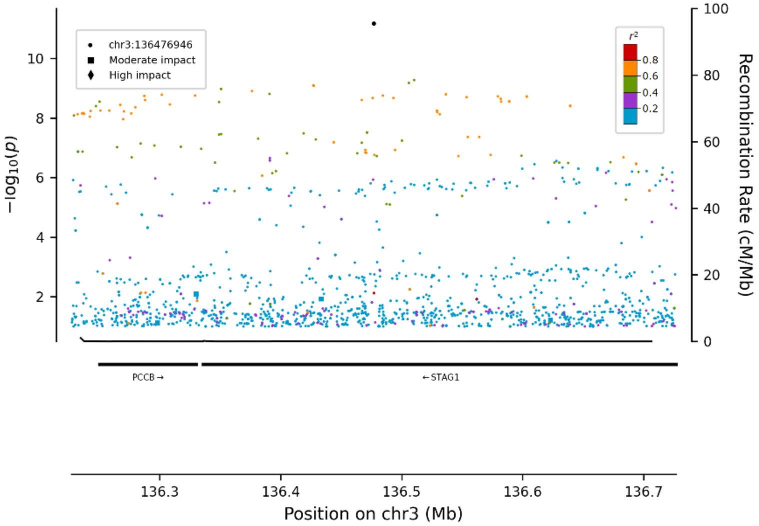
chr3:136476946
Moderate impact
High impact
r^2
0.8
0.6
0.4
0.2
$-\log_{10}(p)$
Recombination Rate (cM/Mb)
PCCB→
←STAG1
136.3
136.4
136.5
136.6
136.7
Position on chr3 (Mb)

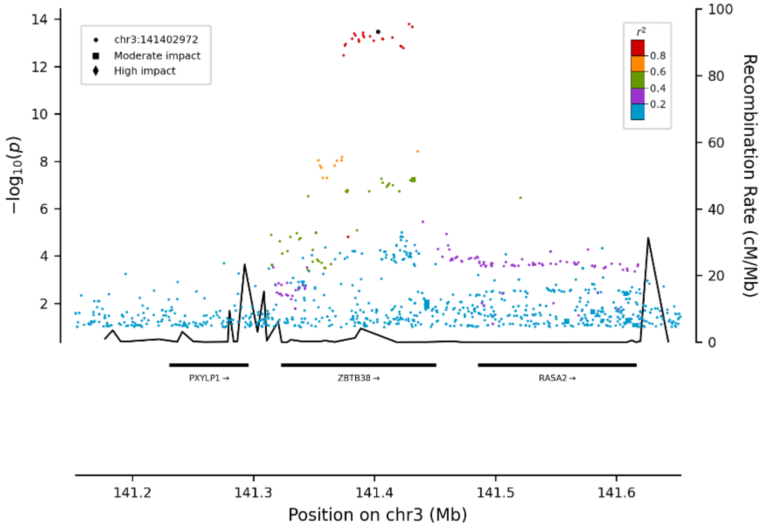
chr3:141402972
Moderate impact
High impact
r^2
0.8
0.6
0.4
0.2
$-\log_{10}(p)$
Recombination Rate (cM/Mb)
PXYLP1→
ZBTB38→
RASA2→
141.2
141.3
141.4
141.5
141.6
Position on chr3 (Mb)

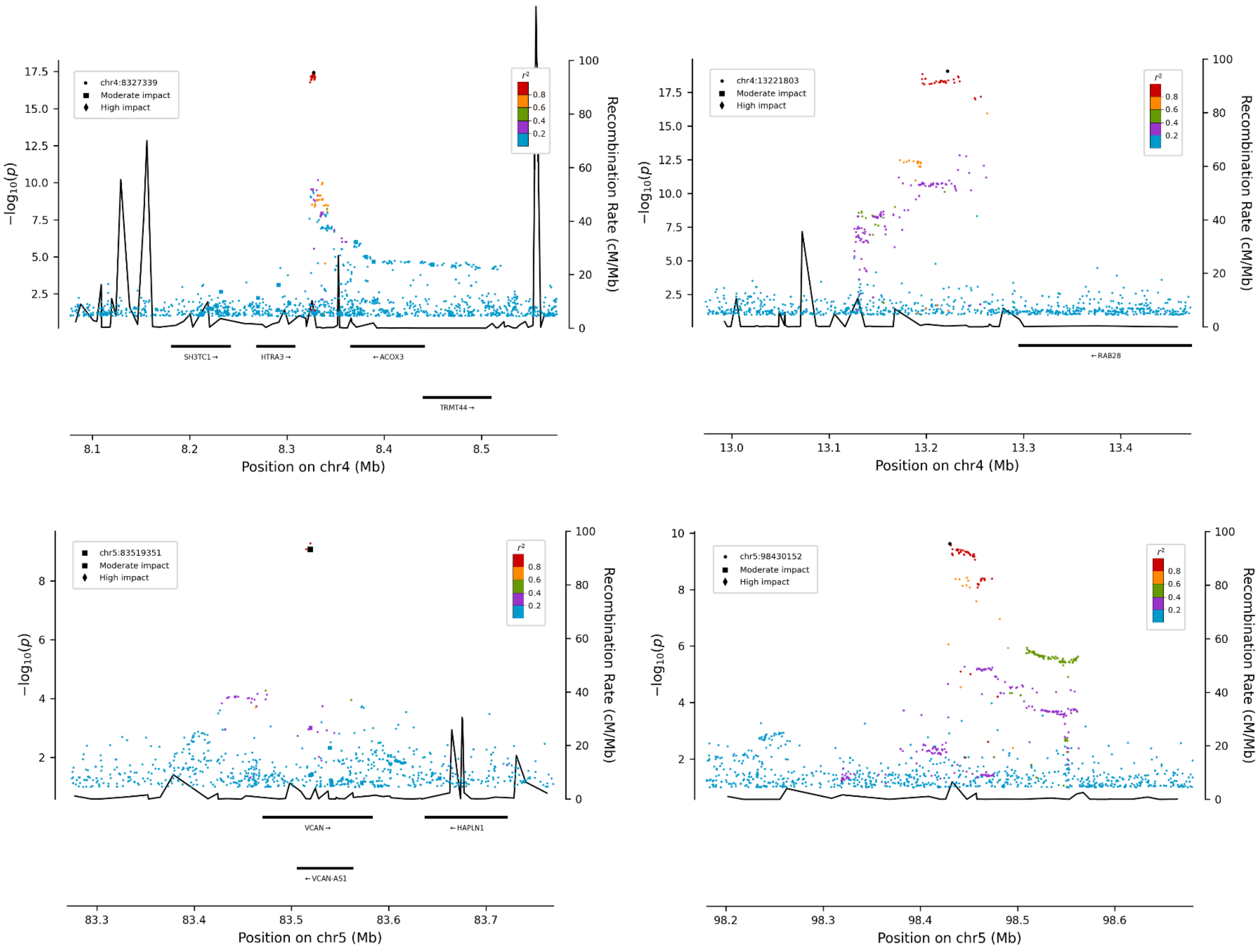
chr4:8327339
Moderate impact
High impact
r^2
SH3TC1→
HTRA3→
←ACOX3
TRMT44→
$-\log_{10}(p)$
Recombination Rate (cM/Mb)
Position on chr4 (Mb)
chr4:13221803
←RAB28
chr5:83519351
VCAN→
←VCAN-AS1
←HAPLN1
Position on chr5 (Mb)
chr5:98430152

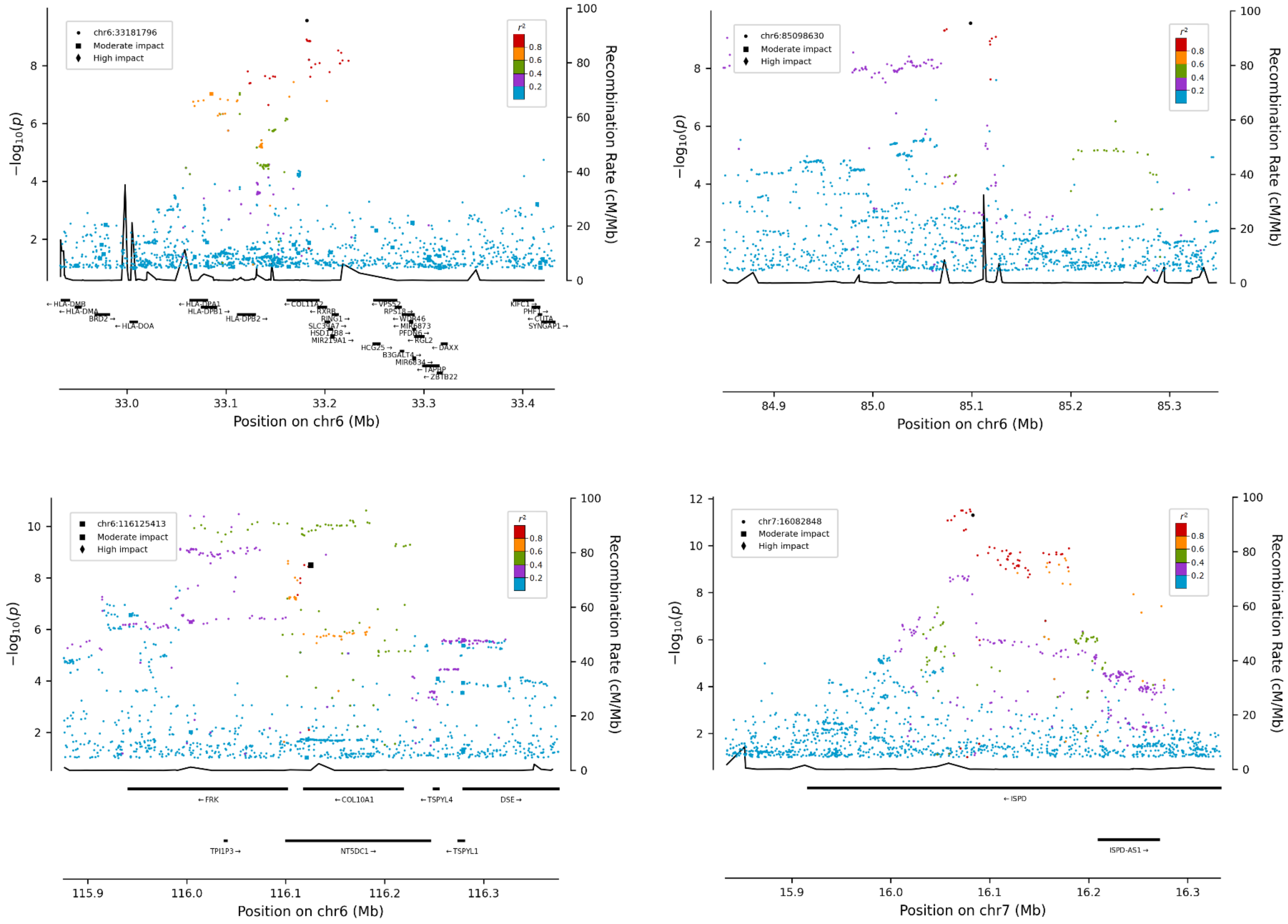
chr6:33181796
Moderate impact
High impact
r^2
0.8
0.6
0.4
0.2
$-\log_{10}(p)$
Recombination Rate (cM/Mb)
Position on chr6 (Mb)
33.0
33.1
33.2
33.3
33.4
chr6:85098630
Position on chr6 (Mb)
84.9
85.0
85.1
85.2
85.3
chr6:116125413
← FRK
← COL10A1
← TSPYL4
DSE →
TPI1P3 →
NT5DC1 →
← TSPYL1
Position on chr6 (Mb)
115.9
116.0
116.1
116.2
116.3
chr7:16082848
← ISPD
ISPD-AS1 →
Position on chr7 (Mb)
15.9
16.0
16.1
16.2
16.3

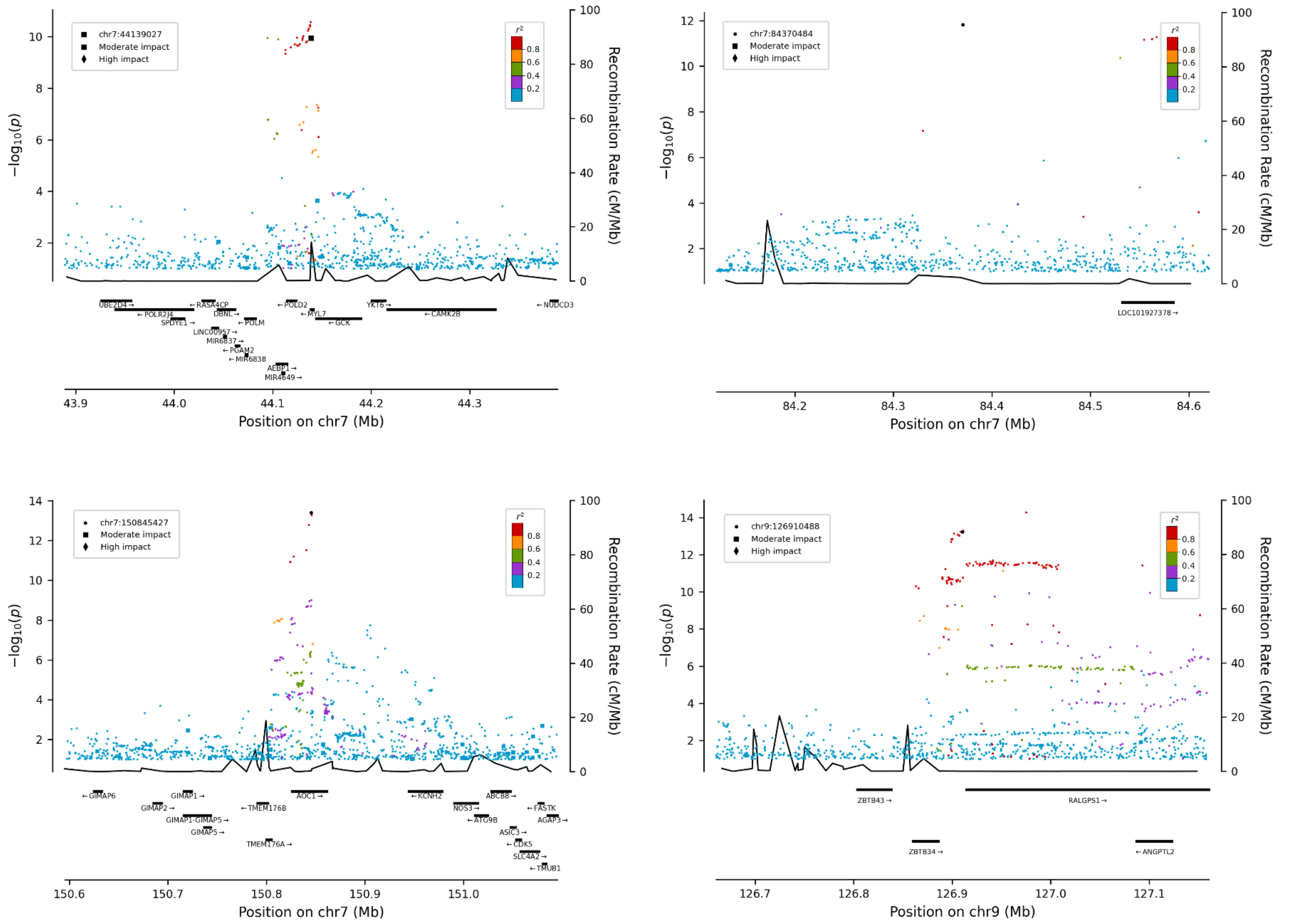

chr7:44139027
Moderate impact
High impact
r^2
Recombination Rate (cM/Mb)
$-\log_{10}(p)$
Position on chr7 (Mb)
UBE2D4→
←POLR2J4
SPDYE1→
←RASA4CP
DBNL→
LINC00957→
MIR6837→
←PGAM2
←MIR6838
←POLM
AEBP1→
MIR4649→
←POLD2
←MYL7
←GCK
YKT6→
←CAMK2B
←NUDCD3
chr7:84370484
LOC101927378→
chr7:150845427
←GIMAP6
GIMAP2→
GIMAP1→
GIMAP1-GIMAP5→
GIMAP5→
←TMEM176B
TMEM176A→
AOC1→
←KCNH2
NOS3→
←ATG9B
ABCB8→
ASIC3→
←CDK5
SLC4A2→
←FASTK
AGAP3→
←TMUB1
chr9:126910488
ZBTB43→
ZBTB34→
RALGPS1→
←ANGPTL2
Position on chr9 (Mb)

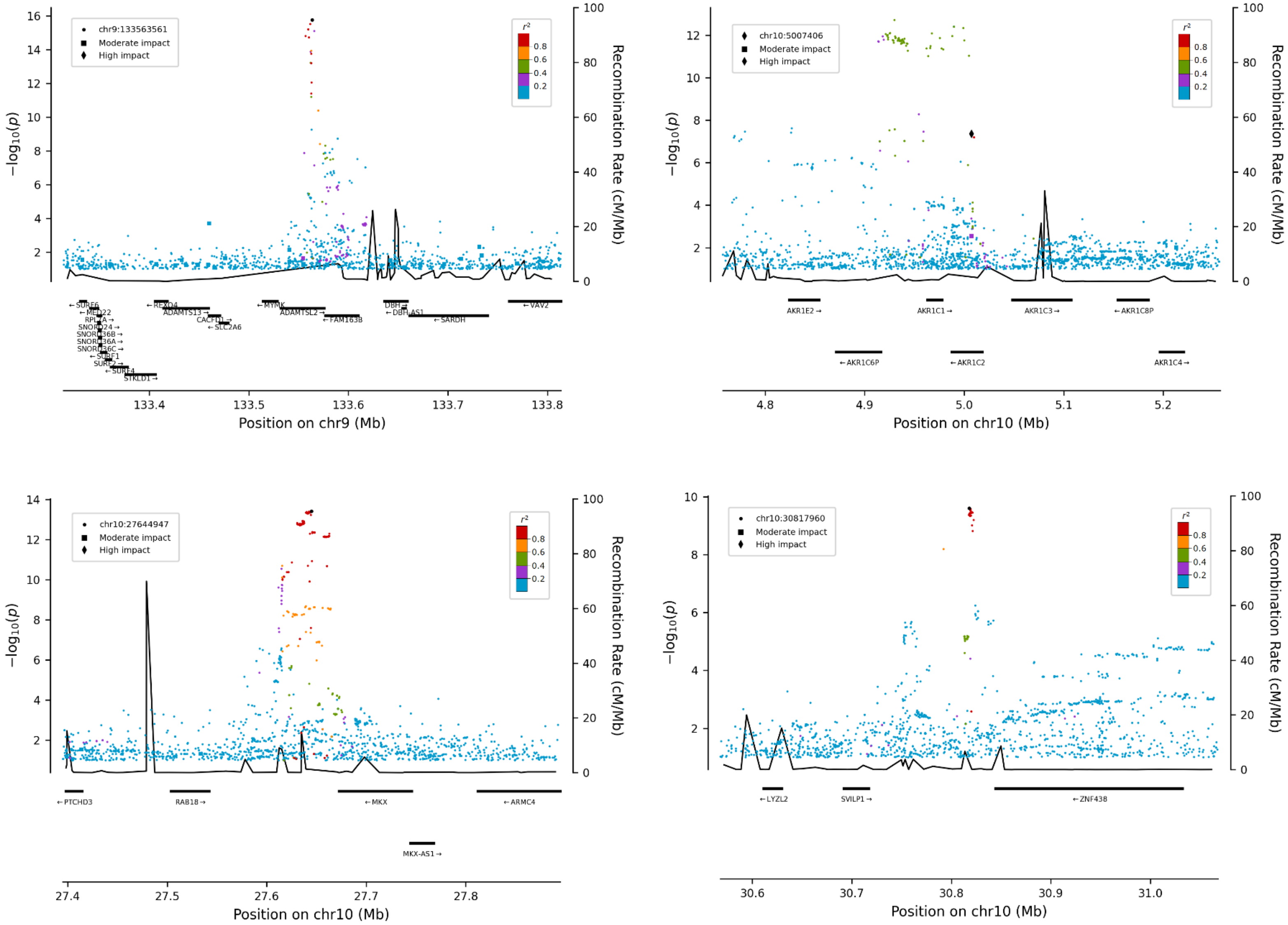
chr9:133563561
Moderate impact
High impact
Position on chr9 (Mb)
Recombination Rate (cM/Mb)
chr10:5007406
Position on chr10 (Mb)
AKR1E2→
AKR1C1→
AKR1C3→
←AKR1C8P
←AKR1C6P
←AKR1C2
AKR1C4→
chr10:27644947
←PTCHD3
RAB18→
←MKX
←ARMC4
MKX-AS1→
chr10:30817960
←LYZL2
SVILP1→
←ZNF438

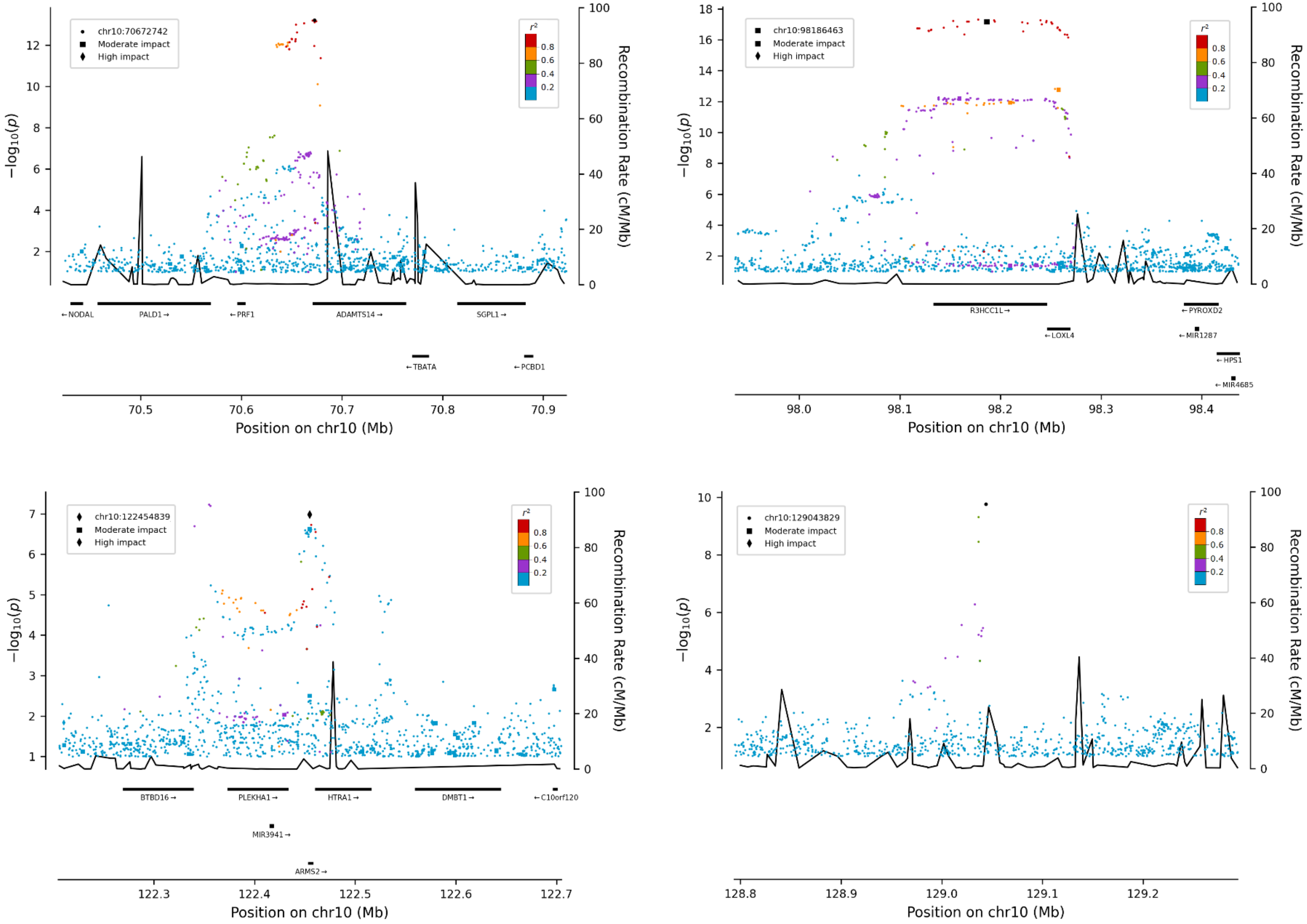
chr10:70672742
Moderate impact
High impact
r^2
Recombination Rate (cM/Mb)
$-\log_{10}(p)$
←NODAL
PALD1→
←PRF1
ADAMTS14→
SGPL1→
←TBATA
←PCBD1
Position on chr10 (Mb)
chr10:98186463
R3HCC1L→
←PYROXD2
←LOXL4
←MIR1287
←HPS1
←MIR4685
chr10:122454839
BTBD16→
PLEKHA1→
HTRA1→
DMBT1→
←C10orf120
MIR3941→
ARMS2→
chr10:129043829

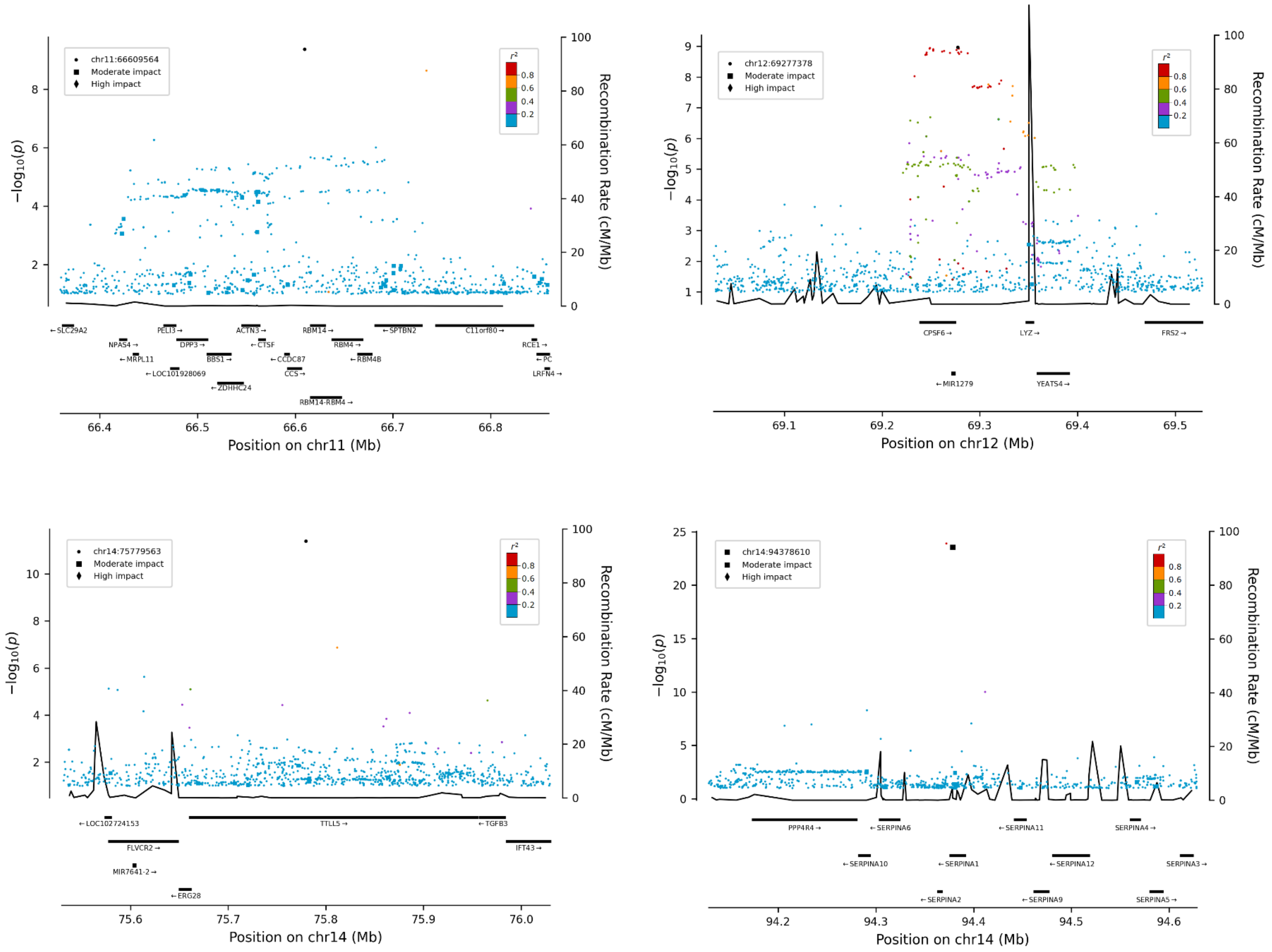
chr11:66609564
Moderate impact
High impact
r^2
0.8
0.6
0.4
0.2
$-\log_{10}(p)$
Recombination Rate (cM/Mb)
←SLC29A2
NPAS4→
←MRPL11
PELI3→
DPP3→
←LOC101928069
BBS1→
←ZDHHC24
ACTN3→
←CTSF
←CCDC87
CCS→
RBM14→
RBM14-RBM4→
RBM4→
←RBM4B
←SPTBN2
C11orf80→
RCE1→
←PC
LRFN4→
66.4
66.5
66.6
66.7
66.8
Position on chr11 (Mb)
chr12:69277378
Moderate impact
High impact
CPSF6→
LYZ→
FRS2→
←MIR1279
YEATS4→
69.1
69.2
69.3
69.4
69.5
Position on chr12 (Mb)
chr14:75779563
Moderate impact
High impact
←LOC102724153
FLVCR2→
MIR7641-2→
←ERG28
TTLL5→
←TGFB3
IFT43→
75.6
75.7
75.8
75.9
76.0
Position on chr14 (Mb)
chr14:94378610
Moderate impact
High impact
PPP4R4→
←SERPINA6
←SERPINA10
←SERPINA1
←SERPINA2
←SERPINA11
←SERPINA12
←SERPINA9
SERPINA4→
SERPINA3→
SERPINA5→
94.2
94.3
94.4
94.5
94.6
Position on chr14 (Mb)

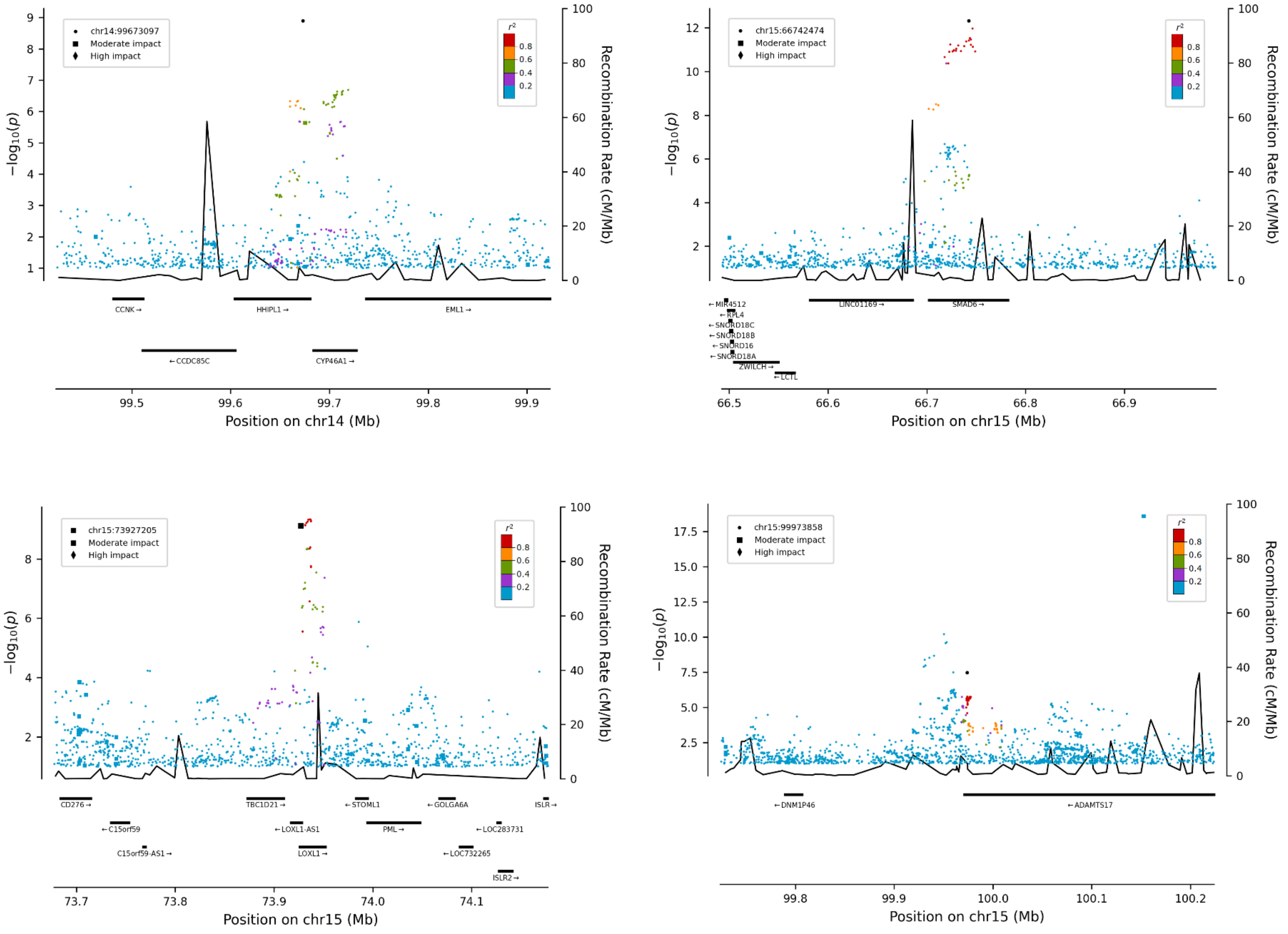
chr14:99673097
Moderate impact
High impact
r^2
−log10(p)
Recombination Rate (cM/Mb)
CCNK→
HHIPL1→
EML1→
←CCDC85C
CYP46A1→
Position on chr14 (Mb)
chr15:66742474
LINC01169→
SMAD6→
←MIR4512
←RPL4
←SNORD18C
←SNORD18B
←SNORD16
←SNORD18A
ZWILCH→
←LCTL
Position on chr15 (Mb)
chr15:73927205
CD276→
TBC1D21→
←STOML1
←GOLGA6A
ISLR→
←C15orf59
←LOXL1-AS1
PML→
←LOC283731
C15orf59-AS1→
LOXL1→
←LOC732265
ISLR2→
Position on chr15 (Mb)
chr15:99973858
←DNM1P46
←ADAMTS17
Position on chr15 (Mb)

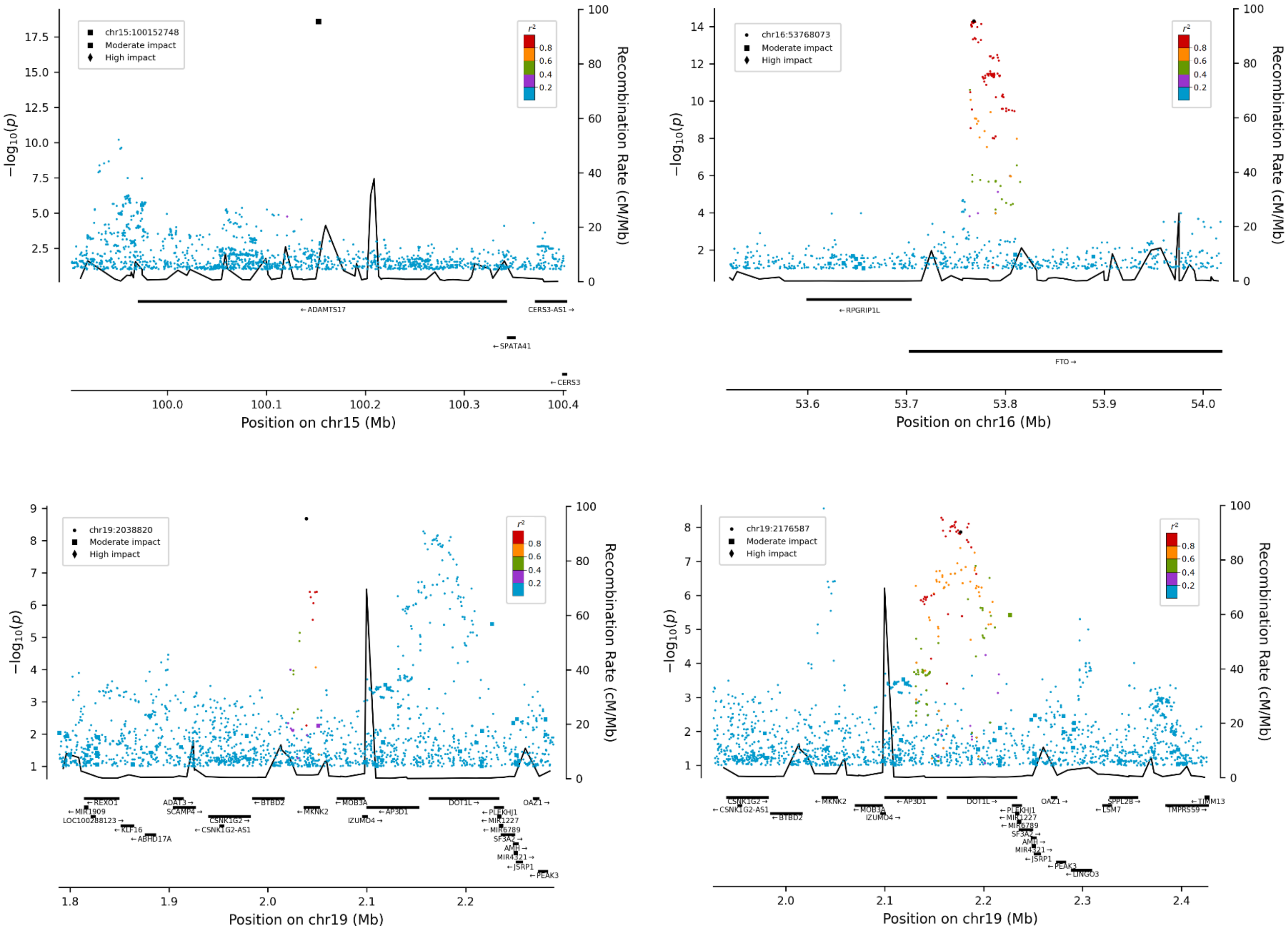
chr15:100152748
Moderate impact
High impact
r^2
$-\log_{10}(p)$
Recombination Rate (cM/Mb)
←ADAMTS17
CERS3-AS1→
←SPATA41
←CERS3
Position on chr15 (Mb)
chr16:53768073
Moderate impact
High impact
←RPGRIP1L
FTO→
Position on chr16 (Mb)
chr19:2038820
Moderate impact
High impact
Position on chr19 (Mb)
chr19:2176587
Moderate impact
High impact
Position on chr19 (Mb)

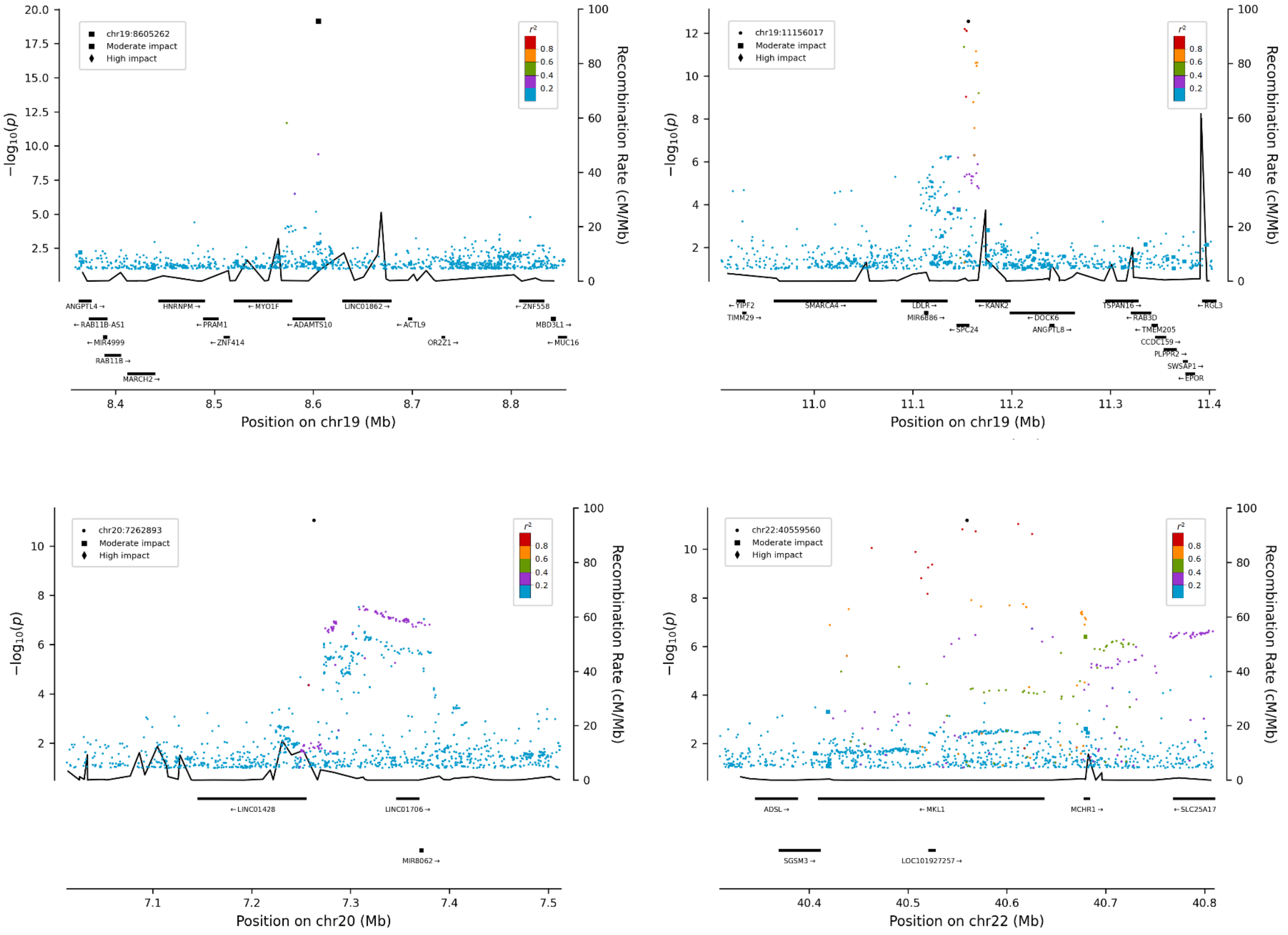
chr19:8605262
Moderate impact
High impact
r^2
0.8
0.6
0.4
0.2
$-\log_{10}(p)$
Recombination Rate (cM/Mb)
ANGPTL4→
←RAB11B-AS1
←MIR4999
RAB11B→
MARCH2→
HNRNPM→
←PRAM1
←ZNF414
←MYO1F
←ADAMTS10
LINC01862→
←ACTL9
OR2Z1→
←ZNF558
MBD3L1→
←MUC16
Position on chr19 (Mb)
chr19:11156017
Moderate impact
High impact
←YIPF2
TIMM29→
SMARCA4→
LDLR→
MIR6886→
←SPC24
←KANK2
←DOCK6
ANGPTL8→
TSPAN16→
←RAB3D
←TMEM205
CCDC159→
PLPPR2→
SWSAP1→
←EPOR
←RGL3
Position on chr19 (Mb)
chr20:7262893
Moderate impact
High impact
←LINC01428
LINC01706→
MIR8062→
Position on chr20 (Mb)
chr22:40559560
Moderate impact
High impact
ADSL→
SGSM3→
←MKL1
LOC101927257→
MCHR1→
←SLC25A17
Position on chr22 (Mb)

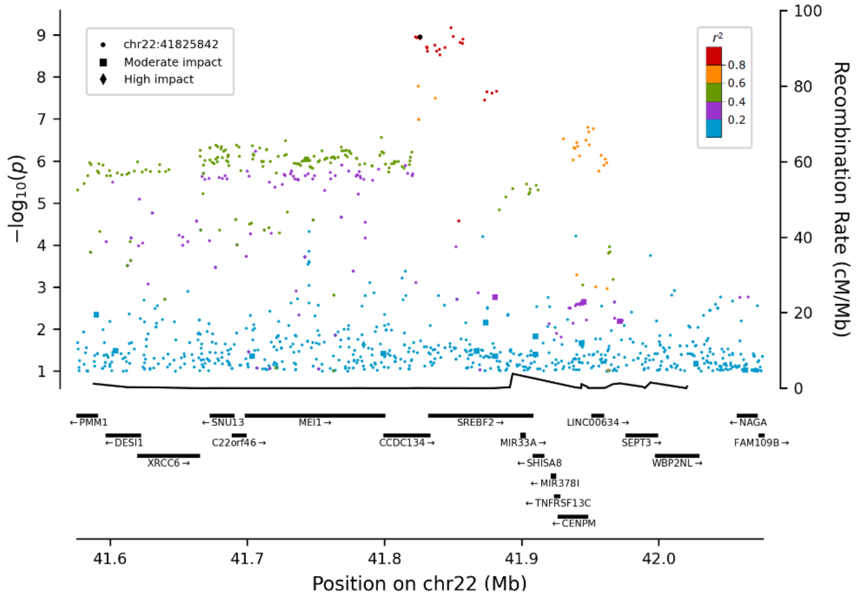

chr22:41825842
Moderate impact
High impact
r^2
0.8
0.6
0.4
0.2
$-\log_{10}(p)$
Recombination Rate (cM/Mb)
←PMM1
←DESI1
XRCC6→
←SNU13
C22orf46→
MEI1→
CCDC134→
SREBF2→
MIR33A→
←SHISA8
←MIR378I
←TNFRSF13C
←CENPM
LINC00634→
SEPT3→
WBP2NL→
←NAGA
FAM109B→
41.6
41.7
41.8
41.9
42.0
Position on chr22 (Mb)

Supplementary Figure 2. Manhattan plot for individual datasets

Manhattan plots showing GWAS results under an additive model for a) Iceland, b) the UK, c) Denmark, and d) Finland. The $-\log_{10}P$-values (y-axis) are plotted for each variant against their chromosomal position (x-axis). *P*-values are two-sided and derived from a likelihood-ratio test.

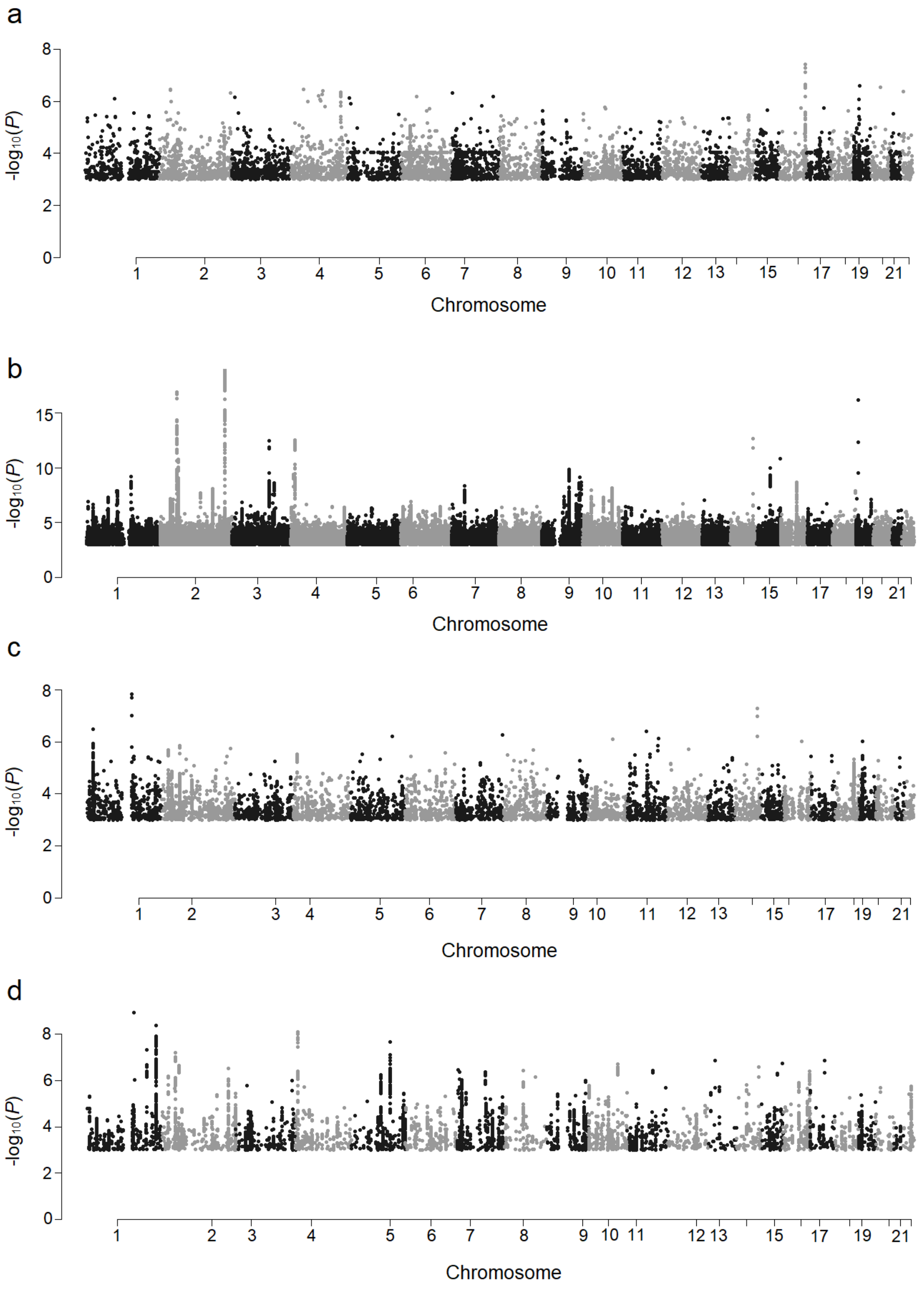

Supplementary Figure 3. Correlation of effects

The correlation of effect sizes for the 53 CTS variants is plotted for the four datasets. The orange line represents results from two-sided weighted linear regression using MAF(1-MAF) as weight. Pearson's correlation coefficient (r) is shown in each plot

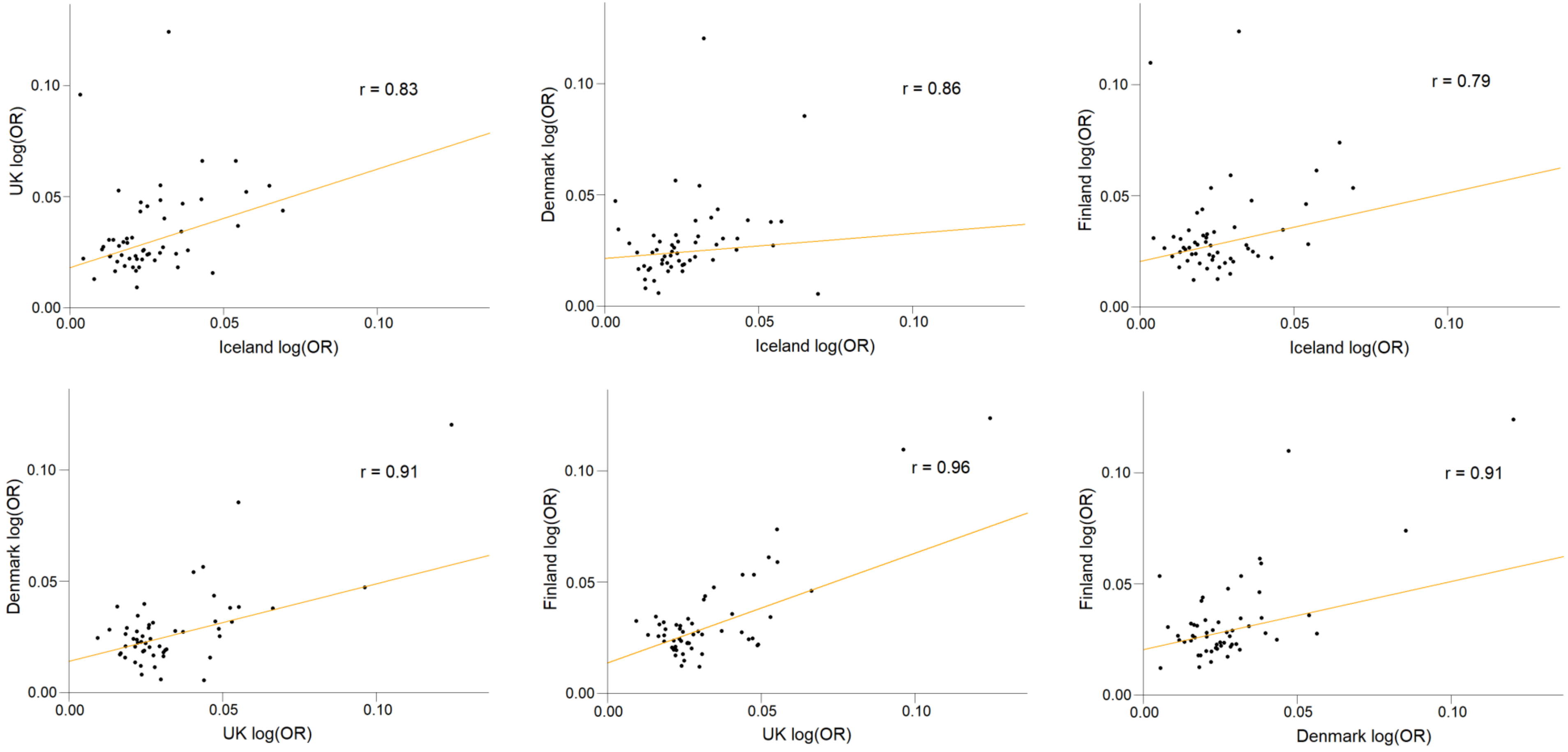

Supplementary Figure 4. Genetic propensity for CTS in different groups

CTS PRSs were constructed for Iceland (navy), The UK (orange), and Denmark (gray). Bilateral, recurrent, or persistent CTS cases had higher PRS than nonrecurrent or nonpersistent cases in all three datasets (Iceland *P*-het = 0.0019, The UK *P*-het = 4.3×10^{-18}, and Denmark *P*-het = 3.6×10^{-4}). The point estimate of OR and 95% CI (error bars) adjusted for genomic inflation on x-axis, were estimated in suggested severity groups, y-axis.

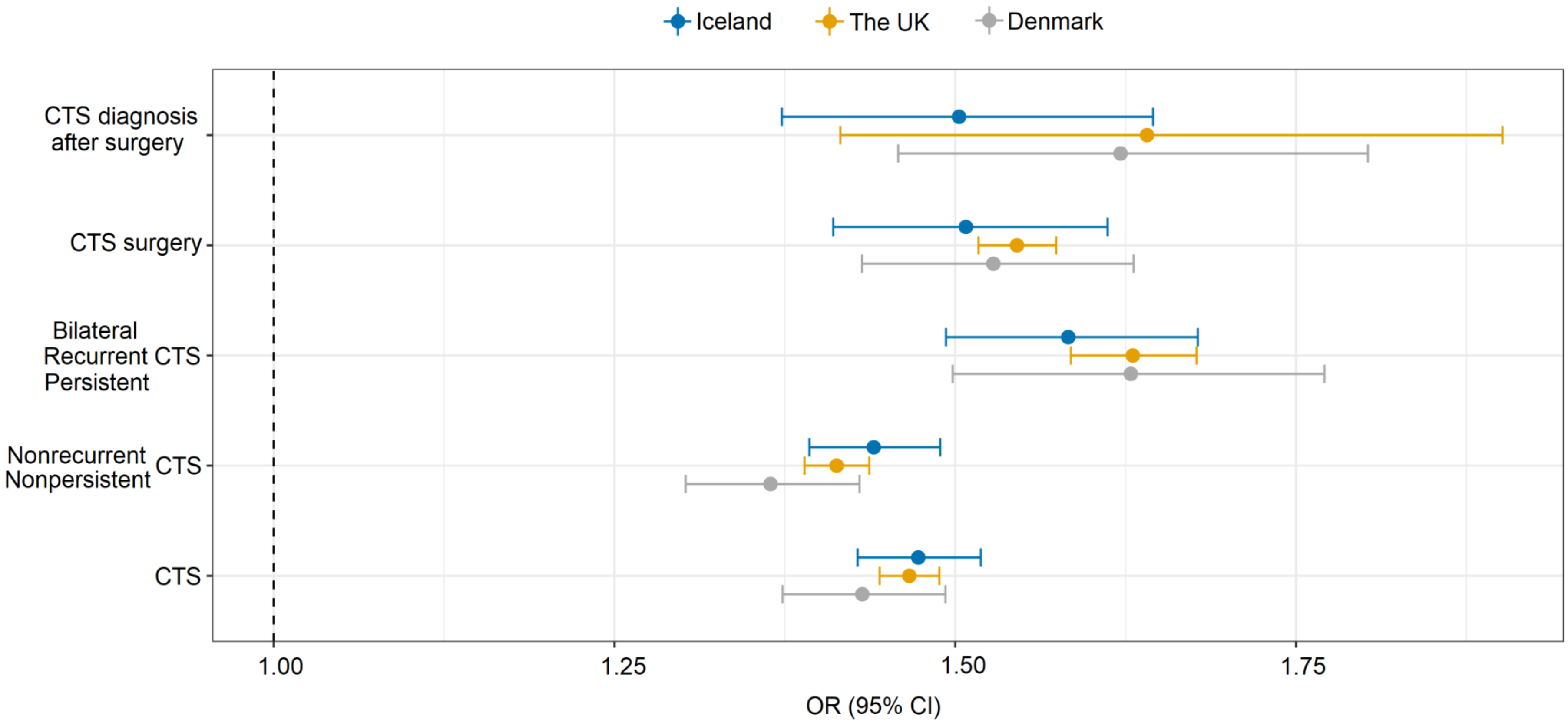

Supplementary Figure 5. Mendelian randomization

Mendelian randomization analyses using inverse variance-weighted (IVW) and MR-Egger methods where the exposure variables were a) height and b) body mass index (BMI) and the outcome variable CTS. We used 3290 variants previously associated with height and 941 variants previously associated with BMI.

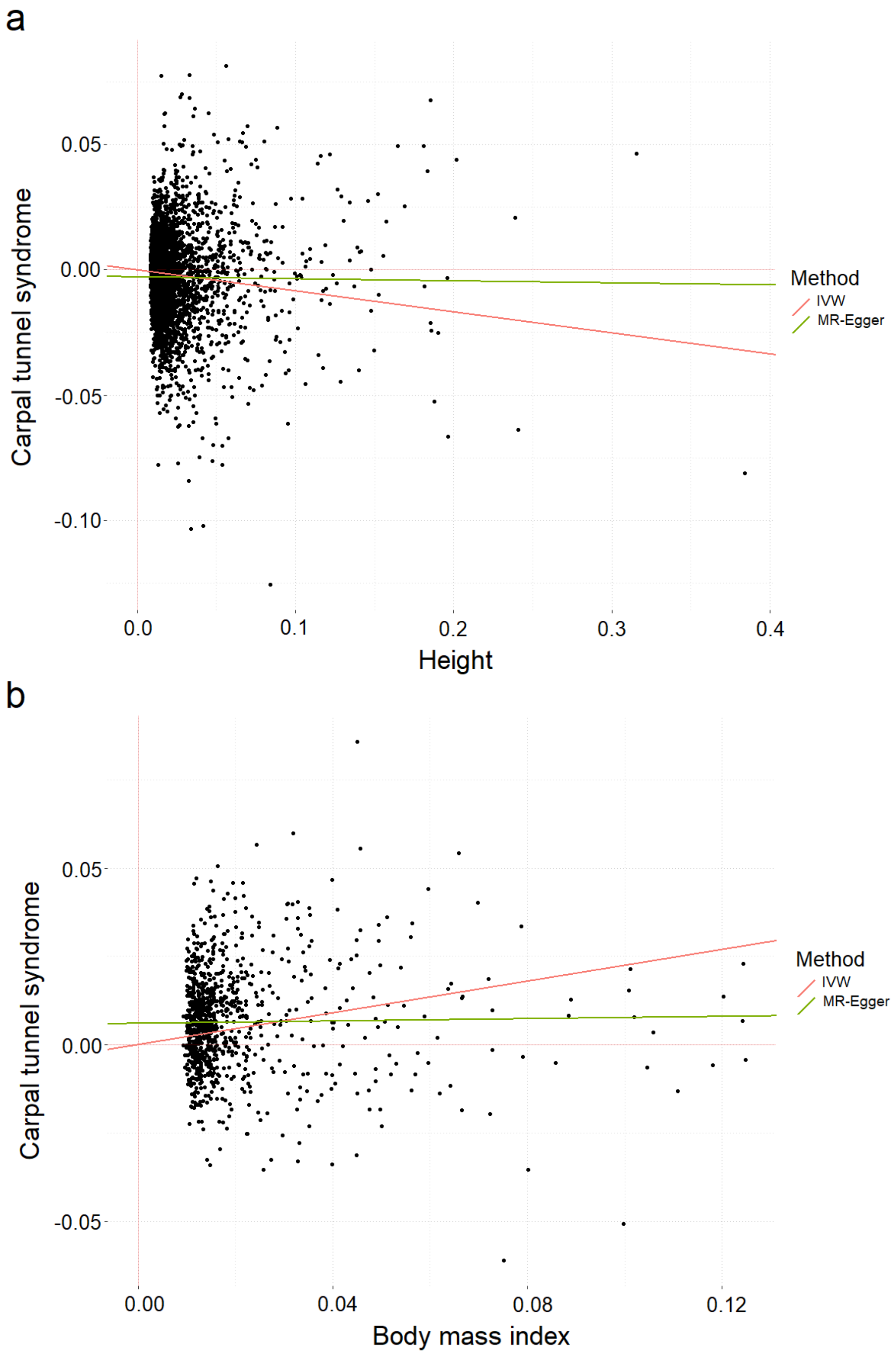

Supplementary Table 1. Demographics of the carpal tunnel syndrome datasets
We had patient onset information for 8077 from Iceland, 18.880 from the UK, and 9323 from Denmark. The bilateral, recurrent, or persistent cases were defined as having at least 6 months in between health encounters. Demographic numbers for the Finnish dataset were accessed at https://r5.risteys.finngen.fi/phenocode/G6_CARPTU.

Dataset	Sex	Count (%)	Mean age at first diagnosis	Median age at first diagnosis	Bilateral, recurrent, or persistent cases (%)
Iceland	Females	5038 (62.0)	51.3	51	23.4
	Males	3084 (38.0)	53.1	53	19.7
The UK	Females	13514 (68.1)	56.3	57	27.2
	Males	6335 (31.9)	59.0	60	25.1
Denmark	Females	5881 (60.9)	55.9	55	28.0
	Males	3783 (39.1)	57.4	58	27.4
Finland	Females	7761 (69.2)	52.5	-	-
	Males	3447 (30.8)	55.4	-	-
Total	Females	32.194 (65.9)	55.1	56	27.1
	Males	16.649 (34.1)	57.1	58	25.4

Supplementary Table 2. Weighted genome-wide significance thresholds

The thresholds were estimated from the Icelandic data. DHS Dnase I hypersensitivity sites

Impact	Type of variants	Broad DHS	Significance threshold
High	Splice donor, splice acceptor, stop gained, frameshift, stop lost, initiator codon	-	1.3×10^{-7}
Moderate	Inframe indels, missense, splice region, stop retained	-	2.6×10^{-8}
Low	Synonymous, 5' UTR, 3' UTR, up- and downstream	-	2.4×10^{-9}
Other	Intronic, intergenic	Yes	1.2×10^{-9}
		No	4.0×10^{-10}

Supplementary Table 3. Mendelian randomization

Mendelian randomization analyses using inverse variance-weighted (IVW) and MR-Egger methods where the exposure variables were a) height and b) body mass index (BMI) and the outcome variable CTS. We used 3290 variants previously associated with height[1] and 941 variants previously associated with BMI[1]. The confidence intervals (CI) were calculated with t-distribution.

Exposure	Method	Estimate	SE	95% CI	*P*-value
Height	IVW	-0.083	0.010	-0.10, -0.064	1.3×10^{-16}
	MR-Egger	-0.008	0.015	-0.037, 0.022	0.60
	Intercept	-0.003	0.000	-0.004, -0.002	1.1×10^{-11}
BMI	IVW	0.23	0.020	0.19, 0.26	3.2×10^{-30}
	MR-Egger	0.016	0.032	-0.045, 0.078	0.604
	Intercept	0.006	0.001	0.005, 0.007	2.2×10^{-16}
