# Supplementary material for: A genome-wide meta-analysis identifies 50 genetic loci associated with carpal tunnel syndrome
Source: Nat Commun. 2022 Mar 24;13:1598. doi: 10.1038/s41467-022-29133-7 (PMC8948232; doi:10.1038/s41467-022-29133-7)
Supplement: Supplementary file 3 — Description of Additional Supplementary Files [file 41467_2022_29133_MOESM3_ESM.pdf]

Description of Additional Supplementary Files

File Name: Supplementary Data 1
Description: Genome-wide associations of CTS meta-analysis. Odds ratios (OR), shown for the effect allele, and P-values for an inverse-variance weighted meta-analysis of association results for the four datasets. Variants were considered significant if they reached weighted genome-wide significance thresholds based on their predicted functional impact. Odds ratios are shown for the effect allele. EA effect allele, OA other allele, EAF effect allele frequency, OR odds ratio

File Name: Supplementary Data 2
Description: Conditional analysis. The analyses were restricted to variants within 1 Mb from the index variants. To account for multiple testing, we chose a conservative P-value threshold of <5E-8 for secondary signals

File Name: Supplementary Data 3
Description: Previously reported associations with CTS and correlated top signals in the CTS meta-analysis

File Name: Supplementary Data 4
Description: Coding variants. The effect allele is reported for the coding variant. EA effect allele, OA other allele

File Name: Supplementary Data 5
Description: cis-eQTL. Variants in LD with the lead variants were identified based on in-house genotype data using r2 > 0.8 for pairwise comparison of the nearest 100,000 variants to define an LD class. We only show eQTL variants that ranked top among all cis-eQTL variants found for a given gene by stepwise conditional association testing. The effect allele is reported for the top-cis eQTL variant. EA effect allele, OA other allele

File Name: Supplementary Data 6
Description: pQTL. Variants in LD with the lead variants were identified based on in-house genotype data using r2 > 0.8 for pairwise comparison of the nearest 100,000 variants to define an LD class. We only show pQTL variants that ranked top among all pQTL variants found for a given gene by stepwise conditional association testing. The effect allele is reported for the top pQTL variant. EA effect allele, OA other allele

File Name: Supplementary Data 7
Description: CADD score to estimate deleteriousness of sequence variants. Variants are considered pathogenic if CADD > 12.37. CADD Combined Annotation Dependent Depletion

File Name: Supplementary Data 8
Description: Gene-set analysis in FUMA. The data was generated on the 2nd of December 2021

File Name: Supplementary Data 9
Description: Genome-wide meta-analysis of lesion of the ulnar nerve. Diagnosis was based on ICD-10 G56.2

File Name: Supplementary Data 10
Description: Genome-wide meta-analysis of lesion of the radial nerve. Diagnosis was based on ICD-10 G56.3

File Name: Supplementary Data 11
Description: Sex-specific model. The variants from the CTS meta-analysis were tested under sex-specific models

File Name: Supplementary Data 12
Description: Genetic correlation. Significant genetic correlations are shown.
