# Supplementary material for: A genome-wide meta-analysis identifies 50 genetic loci associated with carpal tunnel syndrome
Source: Nat Commun. 2022 Mar 24;13:1598. doi: 10.1038/s41467-022-29133-7 (PMC8948232; doi:10.1038/s41467-022-29133-7)
Supplement: Supplementary file 5 — Reporting Summary [file 41467_2022_29133_MOESM5_ESM.pdf]

nature portfolio

Corresponding author(s): Astros Thorarensen Skuladottir
Kari Stefansson

Last updated by author(s): Feb 18, 2022

Reporting Summary

Nature Portfolio wishes to improve the reproducibility of the work that we publish. This form provides structure for consistency and transparency in reporting. For further information on Nature Portfolio policies, see our Editorial Policies and the Editorial Policy Checklist.

Statistics

For all statistical analyses, confirm that the following items are present in the figure legend, table legend, main text, or Methods section.

n/a	Confirmed	
☐	☒	The exact sample size (n) for each experimental group/condition, given as a discrete number and unit of measurement
☐	☒	A statement on whether measurements were taken from distinct samples or whether the same sample was measured repeatedly
☐	☒	The statistical test(s) used AND whether they are one- or two-sided<br>*Only common tests should be described solely by name; describe more complex techniques in the Methods section.*
☐	☒	A description of all covariates tested
☐	☒	A description of any assumptions or corrections, such as tests of normality and adjustment for multiple comparisons
☐	☒	A full description of the statistical parameters including central tendency (e.g. means) or other basic estimates (e.g. regression coefficient) AND variation (e.g. standard deviation) or associated estimates of uncertainty (e.g. confidence intervals)
☐	☒	For null hypothesis testing, the test statistic (e.g. F, t, r) with confidence intervals, effect sizes, degrees of freedom and P value noted<br>*Give P values as exact values whenever suitable.*
☒	☐	For Bayesian analysis, information on the choice of priors and Markov chain Monte Carlo settings
☒	☐	For hierarchical and complex designs, identification of the appropriate level for tests and full reporting of outcomes
☐	☒	Estimates of effect sizes (e.g. Cohen's d, Pearson's r), indicating how they were calculated

Our web collection on statistics for biologists contains articles on many of the points above.

Software and code

Policy information about availability of computer code

Data collection	No software was used for data collection
Data analysis	We used the following publicly available software to analyze the data:<br><br>GraphTyper (v2.0-beta, GNU GPLv3 license) at https://github.com/DecodeGenetics/graphtyper<br><br>Svimmer (v0.1, GNU GPLv3 license), the structural variant merging software at https://github.com/DecodeGenetics/svimmer<br><br>SHAPEIT4 (v4.2.2) at https://odelaneau.github.io/shapeit4/<br><br>Eagle2 (v2.4.1) at http://www.hsph.harvard.edu/alkes-price/software/<br><br>GCTA (v1.93.3beta2) at https://yanglab.westlake.edu.cn/software/gcta/#Overview<br><br>LDPred (v1.0.8) at https://github.com/bvilhjal/ldpred<br><br>LD score regression (first release) at https://github.com/bulik/ldsc<br><br>qqman package (v0.1.6) at https://github.com/stephenturner/qqman<br><br>MendelianRandomization package (v0.5.1) at https://github.com/cran/MendelianRandomization

metafor package (v3.0-2) at https://wviechtb.github.io/metafor/

Axiom genotyping algorithm (v1) at https://www.thermofisher.com/is/en/home.html

FUMA at https://fuma.ctglab.nl/

We used version 3.6.3 of R and version 1.2.5042 of RStudio for statistical analyses and graphs and figures.

For manuscripts utilizing custom algorithms or software that are central to the research but not yet described in published literature, software must be made available to editors and reviewers. We strongly encourage code deposition in a community repository (e.g. GitHub). See the Nature Portfolio guidelines for submitting code & software for further information.

Data

Policy information about availability of data

All manuscripts must include a data availability statement. This statement should provide the following information, where applicable:

- Accession codes, unique identifiers, or web links for publicly available datasets
- A description of any restrictions on data availability
- For clinical datasets or third party data, please ensure that the statement adheres to our policy

The GWAS summary statistics for the CTS meta-analysis are available at https://www.decode.com/summarydata/. Other data generated or analyzed in this study are included in the article and its Supplementary data and information. The study was conducted using the UK Biobank resource under application number 24898 and summary statistics from Finngen for carpal tunnel syndrome was downloaded on May 11th, 2021 from a source available to consortium partners (version 5; http://r5.finngen.fi).

Field-specific reporting

Please select the one below that is the best fit for your research. If you are not sure, read the appropriate sections before making your selection.

☒ Life sciences ☐ Behavioural & social sciences ☐ Ecological, evolutionary & environmental sciences

For a reference copy of the document with all sections, see nature.com/documents/nr-reporting-summary-flat.pdf

Life sciences study design

All studies must disclose on these points even when the disclosure is negative.

Sample size	The sample size was determined by combining all available subjects that had been diagnosed with carpal tunnel syndrome in the four available datasets.
Data exclusions	We excluded variants with imputation information below 0.8 and MAF below 0.01% for quality reasons.
Replication	Our study is a meta-analysis of GWAS results for carpal tunnel syndrome where we combined cases and controls from four populations. Thus, there was no direct replication involved. However, we studied associations of 21 previously reported variants associating in data from the UK, Finland, and Japan. We studied these associations in a meta-analysis of Icelandic and Danish data, excluding the UK and Finnish dataset to avoid potential overlap. One of the variants was a rare variant only found in the Finnish dataset and could not be validated. Of the remaining 20 variants, we found support for 19.
Randomization	No randomizations were used in the study as it is based on a GWAS meta-analysis. The data collected was based on individuals with ICD-10 codes G56.0 in hospital registries. Participation in studies or surveys, particularly genetic studies, is biased toward those wanting to participate (https://www.biorxiv.org/content/10.1101/2022.02.11.480067v1). Randomizing an already biased sample will have a limited effect.
Blinding	This is an observational association study and no blinding was required.

Reporting for specific materials, systems and methods

We require information from authors about some types of materials, experimental systems and methods used in many studies. Here, indicate whether each material, system or method listed is relevant to your study. If you are not sure if a list item applies to your research, read the appropriate section before selecting a response.

Materials & experimental systems

n/a	Involved in the study
☒	☐ Antibodies
☒	☐ Eukaryotic cell lines
☒	☐ Palaeontology and archaeology
☒	☐ Animals and other organisms
☐	☒ Human research participants
☒	☐ Clinical data
☒	☐ Dual use research of concern

Methods

n/a	Involved in the study
☒	☐ ChIP-seq
☒	☐ Flow cytometry
☒	☐ MRI-based neuroimaging

Human research participants

Policy information about studies involving human research participants

Population characteristics	In this study we used subjects diagnosed with carpal tunnel syndrome in Iceland, the UK, Denmark, and Finland that have participated in a research program at deCODE genetics, the UK Biobank, Copenhagen Hospital Biobank, Danish Blood Donor Study, or Finngen. In the study sample, 34% were males and 66% females. The average age at diagnosis was 57 years for males and 55 years for females and the recurrence rate (within 6 months) was 25.4% for males and 27.1% for females.
Recruitment	A large fraction of the Icelandic population (360,000) has participated in a nationwide research program at deCODE genetics. The Icelandic cases in this study were obtained in collaboration with Icelandic physicians at Landspitali – National University Hospital in Reykjavik, the Registry of Primary Health Care Contacts, and the Registry of Contacts with Medical Specialists in Private Practice. We used International Classification of Diseases 10 (ICD-10) code G56.0, ICD-9 code 354.0, and Nomseco Classification of Surgical Procedures (NCSP) code ACC51 (decompression and freeing of nervus medianus) to identify the cases through the scrutiny of hospital records from 1985 to 2020. The cases with lesions of the ulnar and radial nerves were identified using ICD-10 code G56.2 and G56.3, respectively.<br><br>We identified the British/Irish carpal tunnel syndrome cases from the UK Biobank by searching for ICD-10 code G56.0 and Classification of Interventions and Procedures (OPCS) codes A651 (carpal tunnel release) and A692 (revision of carpal tunnel release) in General Practice clinical event records (Field ID 42040) and UK hospital diagnoses (Field ID 41270 and 41271).<br><br>We identified Danish carpal tunnel syndrome cases from Copenhagen Hospital Biobank and Danish Blood Donor Study by searching for ICD-10 code G56.0 and NCSP code ACC51.<br><br>Finnish subjects were identified by Finngen using ICD-10 code G56.0 and ICD-9 code 354.0
Ethics oversight	The Icelandic data in this study was approved by the NBC (VSN-19-158; VSNb2019090003/03.01) following review by the Icelandic Data Protection Authority.<br><br>The North West Research Ethics Committee reviewed and approved UK Biobank's scientific protocol and operational procedures (REC Reference Number: 06/MRE08/65). This study was conducted using the UK Biobank resource under application number 24898.<br><br>The Danish Data Protection Agency (P-2019-51) and the National Committee on Health Research Ethics (NVK-18038012) approved the "Genetics of pain and degenerative diseases" protocol from the Copenhagen Hospital Biobank.<br>The Danish Data Protection Agency (P-2019-99) and the National Committee on Health Research Ethics (NVK-1700407) approved the studies under which genetic data on The Danish Blood Donor Study participants were obtained.<br><br>The Finngen data was approved by The Coordinating Ethics Committee of the Helsinki and Uusimaa Hospital Distric.

Note that full information on the approval of the study protocol must also be provided in the manuscript.
